# Genome-wide association study comparison analysis based on Hanwoo full-sib family

**DOI:** 10.5713/ab.24.0303

**Published:** 2024-06-25

**Authors:** Ji-Yeong Kim, Eun-Ho Kim, Ho-Chan Kang, Cheol-Hyun Myung, Il-Keun Kong, Hyun-Tae Lim

**Affiliations:** 1 Department of Animal Science, Gyeongsang National University, Jinju 52828, Korea; 2 Division of Applied Life Science (BK21 Four), Gyeongsang National University, Jinju 52828, Korea; 3 Institute of Agriculture and Life Science, Gyeongsang National University, Jinju 52828, Korea

**Keywords:** Carcass Traits, Family-based GWAS, Genome-wide Association Study (GWAS), Hanwoo

## Abstract

**Objective:**

The improvement of carcass traits is essential for the Hanwoo industry because of the Hanwoo grade determination system, and genome-wide association study (GWAS) analysis is an instrumental tool for identifying the genetic factors that impact these traits. While GWAS analysis utilizing family data offers advantages in minimizing genetic bias, research on family-based GWAS in Hanwoo is currently lacking.

**Methods:**

This study classified Group A using both parental and offspring genetic information, and Group B based solely on offspring genetic information, to compare GWAS analysis results of Hanwoo carcass traits.

**Results:**

A total of 16 significant single nucleotide polymorphism (SNP) markers were identified in Group A, comprising 7 for carcass weight (CWT), 3 for back fat thickness (BFT), and 6 for marbling score (MS). In Group B, 7 significant SNP markers were identified, including 3 for CWT, 1 for eye muscle area, 1 for BFT, and 2 for MS. Functional annotation analysis revealed only one common function related to carcass traits between the groups, while protein-protein interaction analysis indicated more gene interactions in Group A. The reliability of estimated values for common SNP markers identified between the groups was higher in Group A.

**Conclusion:**

GWAS analysis utilizing parental genetic information holds greater potential for application, owing to its higher reliability of estimated values and the ability to explore numerous candidate genes.

## INTRODUCTION

Hanwoo is a unique breed of *bos taurus* native to Korea, which was widely utilized in the past for agriculture, transportation, rituals, and meat consumptions. However, since meat consumption has risen due to the increase in national income and Hanwoo consumption per capita has significantly increased from 11.3 kg in 2012 to 14.8 kg in 2022, it is currently used in meat production [1,2]. Due to the gentle temperament, adaptability to temperatures, and high productivity of Hanwoo, trait improvement has been ongoing since 1960 under the lead of government and institutions, and the improvement is being carried out in alignment with consumer preferences in meat consumption [1,3]. Hanwoo has been improved by annually selecting approximately 30 superior sires through progeny testing, producing semen from these sires, distributing it to farms, and performing artificial insemination. Thus, in the case of Hanwoo, the Korean proven bull (KPN) is used as a sire, forming a group that considers both pedigree and breeding values. The carcass weight (CWT) has increased from 423.7 kg in 2017 to 430.2 kg in 2022, and marbling score (MS) has increased from 2.0 points to 2.2 points, and consequently, occurrence rate of carcasses with a quality of grade 1 or higher increased by 3.5% and occurrence rate of carcasses with a yield grade of A or B increased by 7.4% in 2022, which indicates that improvement of Hanwoo is directly linked to the improvement of grades [4,5].

The continuous improvement of carcass traits directly affecting the income of Hanwoo farmers is essential [6,7], and traits that take a long time to measure phenotypes have been improved by selecting individuals based on genetic factors to predict the individual ability early on for better selection [8–10]. Recently, the development of commercial chips using single nucleotide polymorphism (SNP) has enabled the rapid obtainment of large-scale genomic information, and the quantity of genome-wide association study (GWAS) research exploring new genetic factors associated with growth and carcass traits through high-density SNP analysis has gradually increased [11–13]. Analysis using the GWAS technique has had a significant impact on identifying genomic variants associated with various diseases or enhanced traits. The analysis primarily focused on specific mutations that affect traits. Among several GWAS-based analyses, family-based GWAS targets groups with relatively close blood relatives, and has been reported to be more effective in complex traits compared to conventional methods, as it reduces bias in genetic effects [14,15]. Although there has been growing interest in utilizing family information in GWAS analysis to remove genetic bias recently [16], the state of family-based GWAS research in Hanwoo is currently insufficient.

Owing to the advancements in biology and genetic engineering technology, GWAS research is evolving towards utilizing gene networks in the analysis of candidate genes for target traits. Since carcass traits of Hanwoo are influenced by polygenic effects, they are regulated by multiple genes, and the analysis utilizing gene networks has shown that genes and the pathway are associated with carcass or meat characteristics [17]. Therefore, through gene ontology (GO) and Kyoto encyclopedia of genes and genomes (KEGG) pathway extraction that assigns functional annotations to genes based on cellular and molecular metabolic processes, the roles of genes and their products can be summarized [18,19]. In addition, protein-protein interaction (PPI) utilizes annotations to easily explain and differentiate the functions of genes, proteins, or their products. By visualizing specific locations or functions of PPI, identifying hub genes that play crucial regulatory roles in the gene and gene expression network associated with the target traits has been streamlined [20,21].

Therefore, this study aimed to discover and investigate SNP markers and candidate genes that affect carcass traits through GWAS targeting the full-sib group, and to compare and analyze the functional annotation analysis (GO, KEGG, PPI) results based on the presence or absence of both paternal and maternal genomic information.

## MATERIALS AND METHODS

### Animal and phenotypic data collection

**Figure 1:**
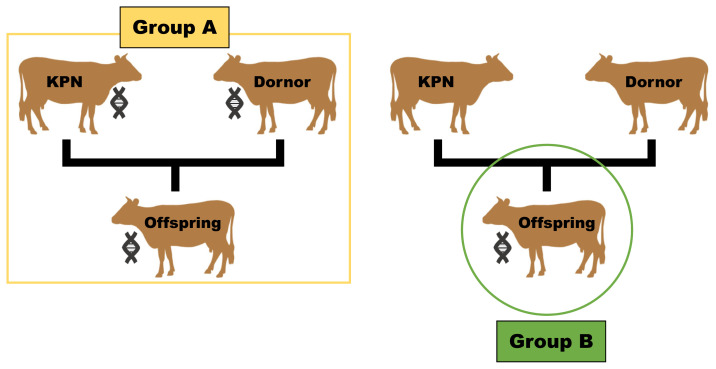
Composition of the analysis groups based on parental genetic information utilization. This diagram differentiates between Group A, which incorporates parental genetic data, and Group B, which relies exclusively on offspring genetic data.

The test group utilized in this study tracked the individual traceability number of Hanwoo produced through embryo transfer from 2017 to 2022, and collected family information by searching the individual transfer numbers at the Korea Animal Improvement Association. To establish the family groups, offspring possessing both maternal and paternal genetic information were considered, while excluding those with outlier carcass performance for analytical convenience. This process resulted in the formation of 15 families, comprising 4 KPN bulls and 14 donor cows, with a total of 374 selected offspring. Each family was defined by having the same parents, and only those families with five or more offspring, from which phenotypic data was obtained through slaughtering, were included in the analysis. Based on the utilization of parental genetic information, the analysis group was categorized into Group A, which included 4 KPN bulls, 14 donor cows, and 374 offspring, and Group B, which only utilized genetic information from the 374 offspring (Figure 1).

Phenotypic information was measured according to the detailed criteria of livestock product grading No. 2014-4 outlined by the Ministry of Agriculture, Food, and Rural Affairs. CWT was measured as the sum of the weights of the left and right cold boning, and the eye muscle area (EMA) was measured by making a perpendicular incision between the left and right thoracic vertebrae and the first lumber vertebra and by measuring the area on the last thoracic vertebra with an area meter. Back fat thickness (BFT) was measured at a point two-thirds along the right side of the EMA towards the abdominal side, and MS was assessed visually by comparing the degree of intramuscular fat deposition at the EMA measurement area with the reference table (1 = Devoid and 9 = abundant).

### SNP genotyping and quality control

A total of 54,609, 53,218, and 53,866 SNP genotypes were obtained using the Bovine SNP50K BeadChip v2, v3, and Hanwoo SNP50K BeadChip v1 (Illumina, San Diego, CA, USA), respectively. For marker selection, quality control was performed using the Plink v1.9 program [22], selecting SNP markers by removing those with a minor allele frequency (MAF) less than 5%, missing genotype exceeding 10%, and Hardy-Weinberg equilibrium (HWE) less than 10^−6^. The number of common SNP markers in three different versions of chips was 45,953, excluding sex chromosomes. In Group A, 34,468 markers were utilized, while 33,816 markers were utilized in Group B.

### GWAS and candidate gene identification

GWAS analysis for each carcass trait was conducted separately by groups using the Univariate Linear Mixed Model option provided by the GEMMA v0.93 program [23]. GEMMA is capable of performing GWAS with adjustments for population and sample structure, and the statistical model is as follows:


$$\begin{array}{l} \text{y}=\text{W}\alpha +\text{x}\beta +\text{u}+ɛ;\\ \text{u}\sim {\text{MVN}}_{\text{n}}\;(0,\lambda \tau^{-1}\text{K}),ɛ\sim {\text{MVN}}_{\text{n}}\;(0,\tau^{-1}{\text{I}}_{\text{n}}) \end{array}$$

In the statistical model, y represents the trait measurement vector, W is the fixed effect covariate matrix of the date of birth, slaughter age, age, and gender, and α is the coefficient vector including the intercept. X is the genotype vector, and β represents the magnitude of the effect of each SNP marker. u is a random effect vector that follows a multivariate normal distribution, where τ^−1^ is the variance of the residual errors, λ represents the ratio between the two variance components, and K represents the genomic relationship matrix calculated with SNP markers of selected autosomes. ε is the residual vector and follows a multivariate normal distribution, and I_n_ represents the identity matrix. A genomic relationship matrix was constructed by incorporating family information, which includes parents and siblings, for each individual.

A suggestive threshold level was used to identify significant markers using the adjusted Bonferroni threshold = 1/number of SNP marker (Group A, p = 2.90×10^−5^; Group B, p = 2.96×10^−5^), and the Bonferroni method = 0.05/number of SNP marker (Group A, p =1.45×10^−6^; Group B, p = 1.48×10^−6^) was applied to confirm the top SNP markers. Candidate genes were explored within 200 kilo bases (kb) upstream and downstream of the SNP marker positions through the National Center for Biotechnology Information gene (NCBI) database ( https://www.ncbi.nih.gov ).

### Functional annotation analysis

The obtained list of candidate genes was utilized for GO analysis using PANTHER [24] based on *Bos taurus* as a reference, and KEGG pathway analysis was conducted utilizing DAVID [25,26]. GO and KEGG pathway was set with a false discovery rate (FDR) of 0.05 or less as confidence, FDR was derived using the Benjamini-Hochberg procedure, and visualization was performed using R software v4.3.1 based on this. The PPI analysis was conducted using the String v12 ( https://string-db.org/) database accessed on July 26, 2023, and the confidence level of PPI was set to 0.4 (median level) based on *Bos taurus*. PPI analysis and visualization were conducted by ensuring that interactions did not exceed 50 based on textmining, experiments, databases, co-expression, neighborhood, gene fusion, and co-occurrence.

## RESULTS AND DISCUSSION

**Figure 2:**
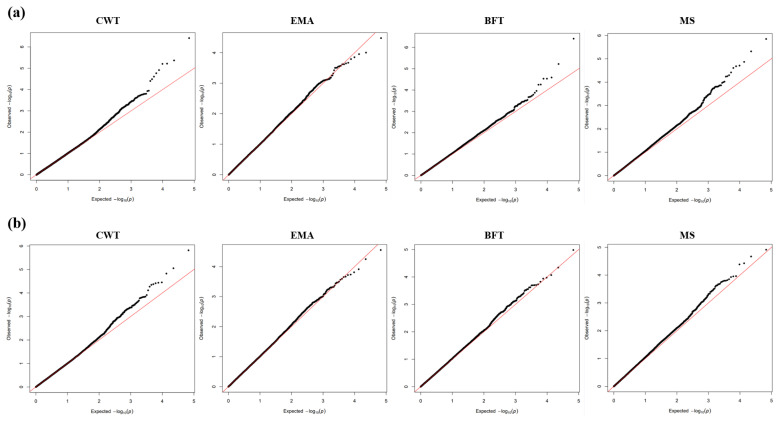
The quantile-quantile (QQ) plots show the distribution of carcass traits by analysis group. The red line indicates the expected log_10_ and the black line indicates the observed log_10_. (a) QQ plot results for Group A. (b) QQ plot results for Group B. CWT, carcass weight; EMA, eye muscle area; BFT, back fat thickness; MS, marbling score.

The basic statistics of carcass trait and phenotype data are shown in Table 1, and the mean and standard deviation of CWT, EMA, BFT, and MS for Group A were 490.28±80.85 kg, 103.96±16.87 cm^2^, 11.71±4.50 mm, and 6.69±2.22 points, respectively. Group B were 493.28±77.59 kg, 104.56±16.28 cm^2^, 11.69±4.19 mm, and 6.84±2.08 points, respectively. When compared to the nationwide Hanwoo carcass grading results in 2022, which were 411.3±76.8 kg, 92.1±15.1 cm^2^, 13.1±5.5 mm, and 5.2±2.3 points, the test group of this study exhibited relatively superior carcass performance [5]. The coefficient of variation is useful for comparing the dispersion between traits with different measurement units, and BFT and MS in this study are more than twice as high compared to CWT and EMA, which confirms a greater phenotypic variability. This is similar to the findings of Nwogwugwu et al [27] and Alam et al [28], where the coefficient of variation for BFT and MS traits in Hanwoo is higher compared to other traits. To assess the bias in the analysis data, a quantile-quantile (QQ) plot was constructed (Figure 2), confirming a normal distribution between the residual quantiles and the predicted quantiles for each carcass trait. This identified the linearity of the two distributions and confirmed that the data points are linearly distributed. Therefore, the data can be considered appropriate for analysis.

**Figure 3:**
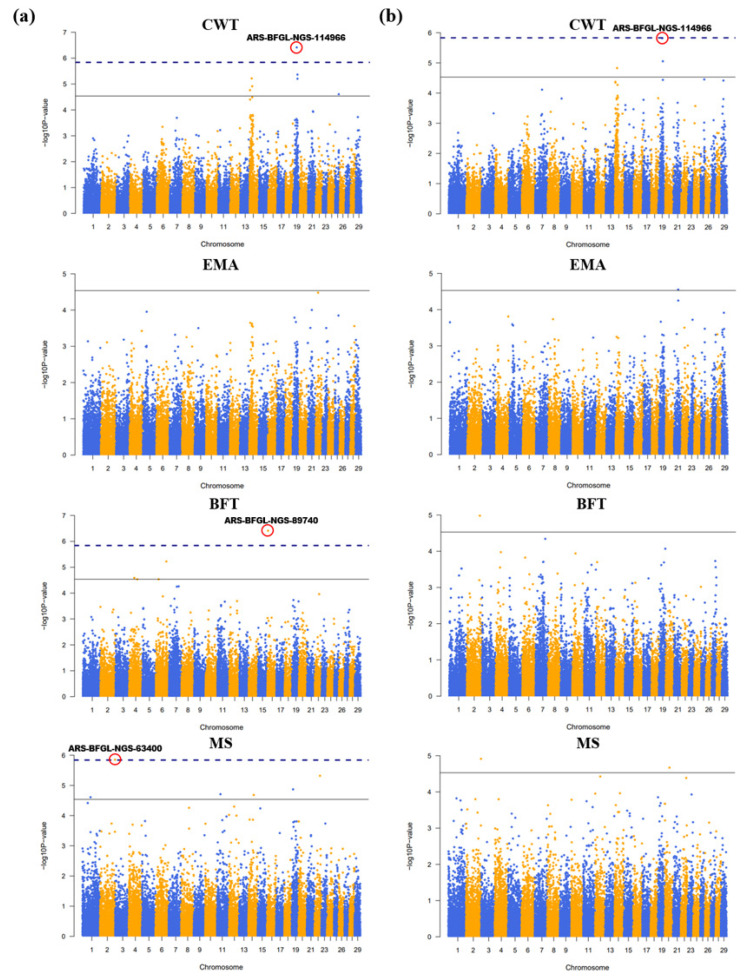
Manhattan plots depicting from the genome-wide association study (GWAS) results by the analysis group. (a) Group A results. (b) Group B results. Significant markers meeting the Bonferroni method are highlighted with red circles, and their corresponding SNP marker names are labeled. CWT, carcass weight; EMA, eye muscle area; BFT, back fat thickness; MS, marbling score.

The linear mixed model is reported to be suitable for utilizing family-based data [29], and SNPs that regulates gene expression and genetic variation of SNP can contribute to phenotypic variability [30]. For the identification of significant SNP markers, the GWAS analysis results were visualized using a Manhattan plot. The Bonferroni method and the Bonferroni threshold differ in how they control the significance level in a statistical hypothesis test. The Bonferroni method divides the significance level by the number of individual hypotheses being tested and applies it to each hypothesis. This approach can result in false negatives and miss important genetic variables. Therefore, the Bonferroni threshold is used to determine the results based on the corrected significance level by reducing the significance level. The black line on the Manhattan plot represents the adjusted Bonferroni threshold, and significant markers were identified by using −log10(p) = 4.54 for Group A and −log10(p) = 4.53 for Group B. The Bonferroni method is indicated by the navy dotted line, and top SNP markers were identified using −log10(p) = 5.84 for Group A and −log10(p) = 5.83 for Group B, and were compared between the groups (Figure 3). In Group A, 7 significant SNP markers were identified for CWT, 3 for BFT, and 6 for MS, and among them 1 marker satisfied the Bonferroni method for each of the CWT, BFT, and MS (Table 2). In Group B, 3 markers were identified for CWT, 1 for EMA, 1 for BFT, and 2 for MS, but only 1 marker for CWT satisfied the Bonferonni method, confirming distinct GWAS analysis results between Group A and Group B. When comparing the p-values of common SNP markers—3 for CWT and 1 for MS—between Group A and Group B, Group A showed more significant estimated values, and the top SNP marker was the same for CWT, but for BFT and MS traits, Group A was only confirmed to have 1 significant marker. Group A and Group B showed divergent results, except for 4 SNP markers, and 16 significant SNP markers were identified in Group A, while 7 were identified in Group B, confirming that utilizing genetic information from both parents and offspring is more useful for exploring genetic factors associated with carcass traits. In Group A, the inclusion of parental genomic information was considered useful for exploring related genetic factors. When comparing both thresholds used in this study, more significant markers were identified in CWT, BFT, and MS, excluding EMA. Therefore, GWAS analysis using parental genomic information is considered useful because it can identify numerous candidate genes associated with complex traits. Additionally, previous studies have reported that the quantitative trait loci (QTL) for CWT were diversely dispersed on chromosomes 14 and 19 [13,31], aligning with the findings of our study. Candidate genes were explored within 200 kb of the SNP marker location [32], identifying a total of 88 candidate genes, with 82 in Group A and 42 in Group B (Table 3). Significant markers for the EMA trait were identified only in Group B. However, candidate genes were selected for CWT, BFT, and MS traits, except for EMA.

*SDCBP* and *TOX*, identified as candidate genes for CWT in Group A, have been reported as candidate genes for CWT of Hanwoo in other studies, but the precise functions and mechanisms has not been revealed [8,33]. *NME1*, identified as a candidate gene for CWT in both Group A and Group B, has been suggested as a gene associated with carcass traits and meat color in pigs [34], but its direct association with BFT and EMA traits could not be confirmed. *ACACA* gene, discovered as a candidate gene for MS in Group A, is a key gene for fatty acid synthesis in mammals and is expressed highly in adipose tissue. It has been reported to be associated with changes in the fatty acid composition of the subcutaneous fat layer in cattle, especially in the longissimus dorsi muscle [35–37]. *TRNAG-CCC* identified in Group B has been suggested to be associated with weight gain in Nellore cattle [38]. *ACTL8* gene that has been commonly identified in the MS of both groups is also a gene involved in muscle growth and development, and it has been reported to be associated with CWT, but its direct association with MS could not be confirmed [39]. The MS candidate gene for Group A—*AATF* and *MANF*—and the MS candidate gene for Group B, the *NPR3*, could not confirm their functions in cattle, but preliminary studies related to adipose tissue were identified. *AATF* is a transcriptional regulatory factor involved in the inhibition of apoptosis [40], and Rodrigues et al [41] reported that apoptosis influences the tenderness of meat. Moreover, while *MANF* deficiency accelerates fat synthesis in mice, *MANF* overexpression inhibits fat synthesis [42]. The expression level of *NPR3* has been reported to significantly increase in obese adults and children, suggesting that it plays a role in the MS of Hanwoo, but further research will be needed [43].

**Figure 4:**
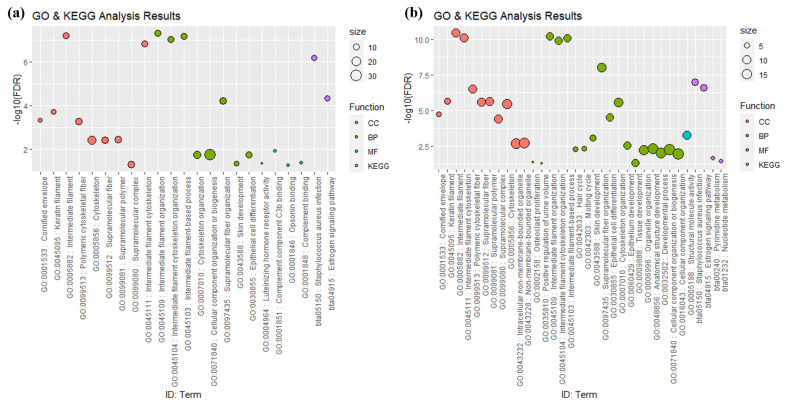
Functional annotation results of GO and KEGG pathway analysis. (a) Group A. (b) Group B. The size of the dot represents the number of genes located in the GO and KEGG databases. GO, gene ontology; BP, biological process; CC, cellular component; MF, molecular function; KEGG, Kyoto encyclopedia of genes and genomes.

Functional annotation analysis, which enables the identification of characteristics and estimated functions of genes and gene products across all organisms, was conducted to verify the biological functions in each group of genes. GO analysis is classified into cellular component (CC), which refers to the internal or external components of a cell, molecular component (MF), which indicates the role of gene products at the molecular level, and biological process (BP), which presents the functional activities of genes in organisms, tissues, and cells. According to the GO analysis, based on FDR<0.05, Group A had a total of 21 functional annotations, consisting of 9 CC, 8 BP, and 4 MF, and Group B had a total of 30 functional annotations, consisting of 11 CC, 18 BP, and 1 MF (Figure 4). The CC and BP in Group A were identified to be common as the CC and BP in Group B, and MF exhibited distinct functionalities in each group. Moreover, Group A and Group B exhibited distinct patterns in GO. Group A had 4 MF, while Group B had 2 CC, 10 BP, and 1 MF. Notably, Group A annotations were predominantly associated with immune responses, whereas Group B annotations were mainly linked to the development and proliferation of cells and organs. In both Group A and Group B, GO: 0005882 of CC and GO: 0045109 of BP were ranked highly. According to the GO analysis results, it predominantly participates in the composition of filament and cellular cytoskeleton, and intermediate filament (GO: 0005882) constitutes cellular cytoskeleton proteins, and has a direct correlation with body weight [44]. A single GO term was confirmed to be commonly associated with carcass traits in both groups. When comparing the FDR of the common GO function identified in Groups A and B, Group B exhibited lower FDR values. However, the difference in p-value and FDR value for the common GO between groups are attributed to the variations in the analysis, resulting from the number of input gene sets in each analysis group during the calculation of the gene list and GO term gene set. Therefore, it can be determined that utilizing the results of GO analysis conducted with a small gene set list is challenging for intergroup comparisons.

In the KEGG analysis, pathways such as ‘Staphylococcus aureus infection’ and ‘Estrogen signaling pathway’ were identified in Group A, and ‘Pyrimidine metabolism’ and ‘Nucleotide metabolism’ were additionally identified in Group B, confirming more gene functions and pathways elucidated in Group B compared to Group A. The KEGG analysis revealed the involvement of sufficient genes in pathways related to disease and immune function, such as the Staphylococcus aureus infection pathway (bta05150) and Estrogen signaling pathway (bta04915), and are predominantly associated with the Keratin genes family, which has been reported to be primarily associated with hair in cattle [45]. Therefore, the pathway analysis groups genes participating in the same BP, but direct pathways specifically associated with the carcass traits in Hanwoo were not identified.

**Figure 5:**
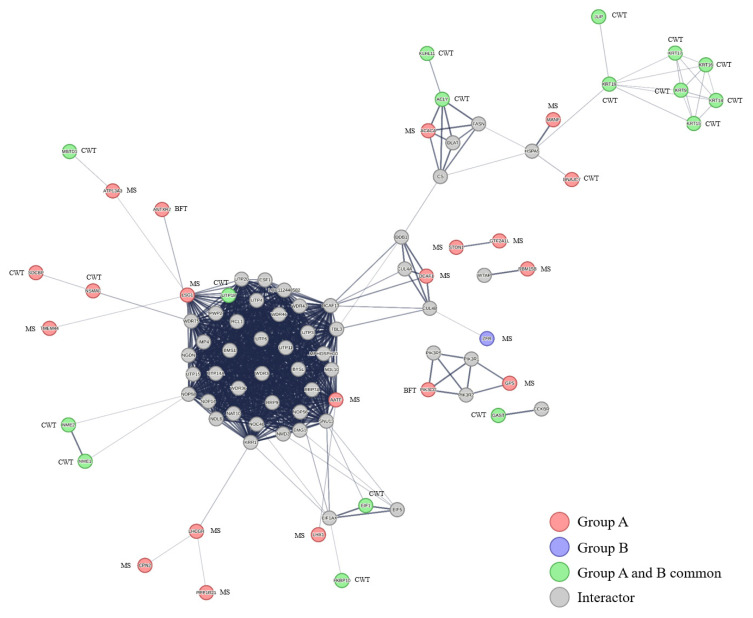
The protein-protein interaction (PPI) network is formed by the candidate genes from groups A and B. The red color indicates candidate genes from group A, the blue color indicates candidate genes from group B, and the green color indicates candidate genes that are common to both groups A and B. The thicker the line, the more interaction it represents. Genes that do not participate in the network were excluded.

PPI analysis was performed for visualization to examine the functions of genes extracted from Go and KEGG (Figure 5). In PPI analysis, interactions refer to the physical interactions between proteins, where proteins either bind to one another or interact to carry out biological functions. These experimentally observed interactions vary based on the proteins’ structure and function and are depicted in a PPI network, where each node symbolizes a protein and each edge represents an interaction between proteins. In this study, 106 nodes and 817 edges were confirmed, and the candidate genes of Group A and Group B were identified to exclude genes that are not involved in the networks from visualization. According to PPI analysis results, Group A was revealed to have more nodes and edges compared to Group B, indicating that the candidate genes of Group A have diverse set of interactions with other genes. Group A can form more complex networks due to having more genes and interactions compared to group B. This highlights the functional diversity of group A. PPI allows to confirm the unidentified interactions among proteins and genes, and can discover the expression mechanisms of proteins [21].

GWAS is a comprehensive analysis technique that allows for the exploration of all genetic variations of SNPs associated with a specific trait, enabling the identification of genetic biomarkers [46]. Family-based GWAS introduces a novel strategy to correct for population structure [14,15]. In this study, we aim to compare analysis results by using genetic information from parents to explore the genetic relationship matrix in families, including the father, mother, and their offspring. According to the comparison analysis between groups based on the utilization of both paternal and maternal genetic information, Group A was found to have a significantly higher number of SNP markers compared to Group B, and the p-values of SNP markers commonly verified were found to be more significant in Group A. The gene function associated with carcass traits was reported more in previous studies for the candidate genes identified in Group A. Although Group B revealed a higher number of functions and pathways in GO and KEGG analysis, only one function related to CWT was commonly identified between the groups in GO. When conducting GWAS analysis to investigate genes and functionalities related to carcass traits, incorporating genetic data from both parents and offspring results in more significant SNP markers. Thus, it is more beneficial for identifying causal genes related to carcass traits. Further analysis with an increased number of typical full-sib family groups is necessary for additional insights.

## CONCLUSION

This study aimed to compare the GWAS analysis results on carcass traits based on the utilization of family information consisting of 15 Hanwoo full-sib family groups. The analysis groups were classified into Group A and Group B based on the utilization of parental genetic information, to distinguish the utilization of both paternal and maternal genetic information. According to the analysis results, Group A identified 16 significant SNP markers (CWT 7, BFT 3, MS 6) and Group B identified 7 significant SNP markers (CWT 3, EMA 1, BFT 1, MS 2), and a total of 88 candidate genes were selected, with 82 in Group A and 42 in Group B. In terms of GO, 1 GO function related to CWT was commonly identified between the two groups with 21 for Group A and 30 for Group B. Pathway was 2 for Group A and 4 for Group B, while PPI analysis showed that Group A had a higher number of edges and nodes. Therefore, GWAS analysis utilizing paternal and maternal genetic information is expected to be useful for identifying SNP markers and candidate genes associated with carcass traits.

## Figures and Tables

**Table 1:** Comparison of test group carcass trait statistics

Group	Trait	Number of animal	Mean	Min	Max	SD	CV (%)
A	CWT (kg)	392	490.28	204	691	80.85	16.49
	EMA (cm^2^)	392	103.96	39	146	16.87	16.23
	BFT (mm)	392	11.71	2	39	4.50	38.47
	MS (1 to 9)	392	6.69	1	9	2.22	33.15
B	CWT (kg)	374	493.28	204	691	77.59	15.73
	EMA (cm^2^)	374	104.56	47	146	16.28	15.57
	BFT (mm)	374	11.69	2	25	4.19	35.84
	MS (1 to 9)	374	6.84	1	9	2.08	30.41

SD, standard deviation; CV, coefficient of variation; CWT, carcass weight; EMA, eye muscle area; BFT, back fat thickness; MS, marbling score.

**Table 2:** Group A and B significant single nucleotide polymorphism is indicated by a black line

Group	Trait	Chr	SNP	Position (bp)	Allele	MAF	p-value
A	CWT	14	ARS-BFGL-NGS-109902	4,984,674	A/G	0.298	1.72×10^−5^
		14	UA-IFASA-6356	20,347,849	A/G	0.458	6.08×0^−6^
		14	rs133832329	24,920,882	G/A	0.488	1.22×10^−5^
		19	ARS-BFGL-NGS-114966	35,555,115	A/G	0.347	3.85×10^−7^
		19	ARS-BFGL-NGS-36183	41,926,734	C/G	0.263	4.31×10^−6^
		19	BTA-123276-no-rs	41,950,232	A/G	0.32	6.25×10^−6^
		25	BTA-101631-no-rs	32,089,261	A/G	0.318	2.48×10^−5^
	BFT	4	BTB-00183304	47,909,532	A/G	0.194	2.58×10^−5^
		6	ARS-BFGL-NGS-94213	94,668,310	G/A	0.258	6.00×10^−6^
		16	ARS-BFGL-NGS-89740	6,168,426	A/G	0.499	3.90×10^−7^
	MS	1	ARS-BFGL-NGS-94206	73,048,145	A/G	0.172	2.46×10^−5^
		2	ARS-BFGL-NGS-63440	134,792,816	A/G	0.165	1.41×10^−6^
		11	ARS-BFGL-NGS-112032	30,968,132	A/G	0.051	1.95×10^−5^
		14	rs133053966	50,206,540	C/A	0.077	2.08×10^−5^
		19	ARS-BFGL-NGS-33447	13,313,876	A/G	0.07	1.35×10^−5^
		22	ARS-BFGL-NGS-26408	49,402,072	A/G	0.051	4.80×10^−6^
B	CWT	14	UA-IFASA-6356	20,347,849	A/G	0.450	1.49×10^−5^
		19	ARS-BFGL-NGS-114966	35,555,115	A/G	0.342	1.53×10^−6^
		19	ARS-BFGL-NGS-36183	41,926,734	C/G	0.261	8.85×10^−6^
	EMA	21	Hapmap39843-BTA-52224	38,948,511	G/A	0.082	2.79×10^−5^
	BFT	2	ARS-BFGL-NGS-30337	116,542,326	A/G	0.166	1.04×10^−5^
	MS	2	ARS-BFGL-NGS-63440	134,792,816	G/C	0.156	1.22×10^−5^
		20	ARS-BFGL-NGS-109580	41,098,240	A/C	0.138	2.14×10^−5^

Chr, chromosome; SNP, single nucleotide polymorphism; bp, base pair; MAF, minor allele frequency; CWT, carcass weight; BFT, back fat thickness; MS, marbling score; EMA, eye muscle area.

**Table 3:** Group A and B positional candidate genes

Group	Trait	Chr	SNP	Genes in the vicinity (±200 kb) of the marker
A	CWT	14	ARS-BFGL-NGS-109902	-
		14	rs133832329	*LOC107133116, NSMAF, SDCBP*^a^, *TOX*^a^
		19	BTA-123276-no-rs	*ACLY, CNP, DNAJC7, EIF1, FKBP10, GAST, HAP1, HAPP, JUP, KLHL10, KLHL11, KRT9, KRT14, KRT15, KRT16, KRT17, KRT19, KRT42, LOC107131524, LOC112441510, LOC112442771, LOC112442825, LOC104975084, NT5C3B, ODAD4, P3H4, TRNAC-GCA*
		25	BTA-101631-no-rs	*-*
	BFT	4	BTB-00183304	*CCDC71L, LOC104972036, LOC104972045, LOC112446374, LOC781229, PIK3CG*
		6	ARS-BFGL-NGS-94213	*ANTXR2*
		16	ARS-BFGL-NGS-89740	*CFH, LOC781004, LOC790886, LOC101905630, LOC101907330*
	MS	1	ARS-BFGL-NGS-94206	*ATP13A3, CPN2, FAM43A, GP5, LOC104970893, LOC104970894, LOC112447381, LOC112447393, LRRC15, LSG1, MIR2287, TMEM44*
		11	ARS-BFGL-NGS-112032	*GTF2A1L, LHCGR, PPP1R21, STON1*
		14	rs133053966	*LOC112449645*
		19	ARS-BFGL-NGS-33447	*AATF*^a^, *ACACA*^a^, *LHX1, LOC507271*
		22	ARS-BFGL-NGS-26408	*DCAF1, DOCK3, LOC112443475, MANF*^a^, *RAD54L2, RBM15B*
B	EMA	21	Hapmap39843-BTA-52224	*-*
	BFT	2	ARS-BFGL-NGS-30337	*LOC514681, LOC781122*
	MS	20	ARS-BFGL-NGS-109580	*NPR3*^a^, *LOC104975283, TRNAG-CCC*^a^, *ZFR*
A, B	CWT	14	UA-IFASA-6356	*LOC511847, SNTG1*
		19	ARS-BFGL-NGS-114966	*LOC107131519, LOC112442573, LOC112442830, MBTD1, NME1*^a^, *NME2, SPAG9, UTP18*
		19	ARS-BFGL-NGS-36183	*ACLY, EIF1, FKBP10, GAST, HAP1, HAPP, JUP, KLHL10, KLHL11, KRT9, KRT14, KRT15, KRT16, KRT17, KRT19, KRT42, LOC107131524, LOC112441510, LOC112442771, LOC112442825, LOC104975084, NT5C3B, ODAD4, P3H4*
	MS	2	ARS-BFGL-NGS-63440	*ACTL8*^a^, *ARHGEF10L*

Chr, chromosome; SNP, single nucleotide polymorphism; kb, kilobase; CWT, carcass weight; BFT, back fat thickness; MS, marbling score; EMA, eye muscle area.

a A gene that has been reported to be associated with carcass traits in previous studies.
